# Supplementary material for: Cellular Toxicity Mechanisms and the Role of Autophagy in Pt(IV) Prodrug-Loaded Ultrasmall Iron Oxide Nanoparticles Used for Enhanced Drug Delivery
Source: Pharmaceutics. 2021 Oct 19;13(10):1730. doi: 10.3390/pharmaceutics13101730 (PMC8541321; doi:10.3390/pharmaceutics13101730)
Supplement: Supplementary file 1 [file pharmaceutics-13-01730-s001.zip › pharmaceutics-1332400-supplementary.pdf]

pharmaceutics

MDPI

Supplementary Material: Cellular Toxicity Mechanisms and the Role of Autophagy in Pt(IV) Prodrug-Loaded Ultrasmall Iron Oxide Nanoparticles Used for Enhanced Drug Delivery

L. Gutiérrez-Romero, L. Rivas, C. González Sánchez, J. Llopis, E. Blanco and M. Montes-Bayón

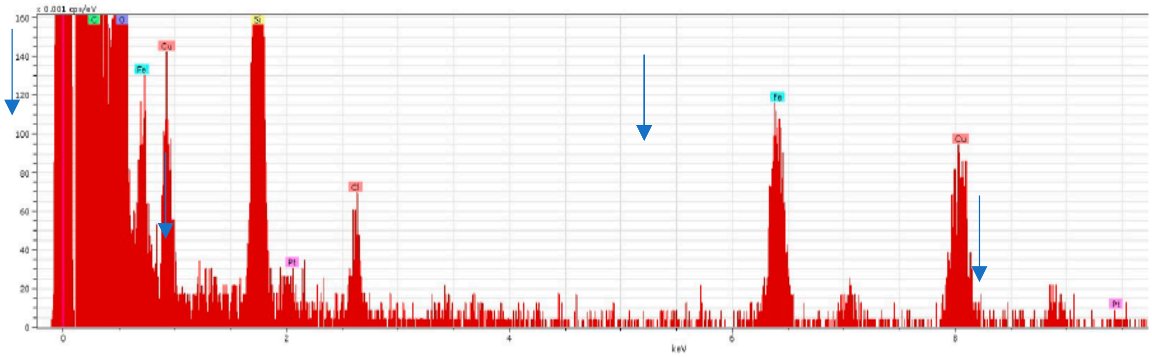

(A)

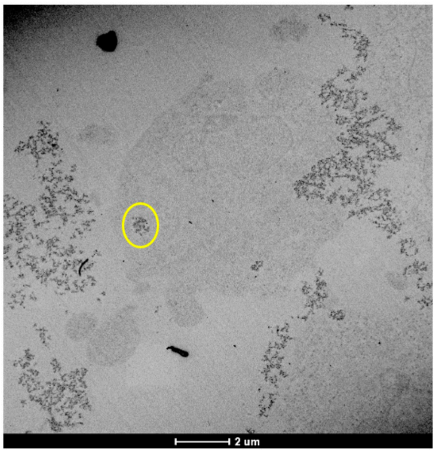

(B)

Figure S1. (**A**) EDX analysis of the nanoparticles observed by (**B**) TEM present within the cell cytosol (arrows pointing the Pt and Fe signals). Since Pt is only present on the surface of the particles, the intensity is significantly smaller.

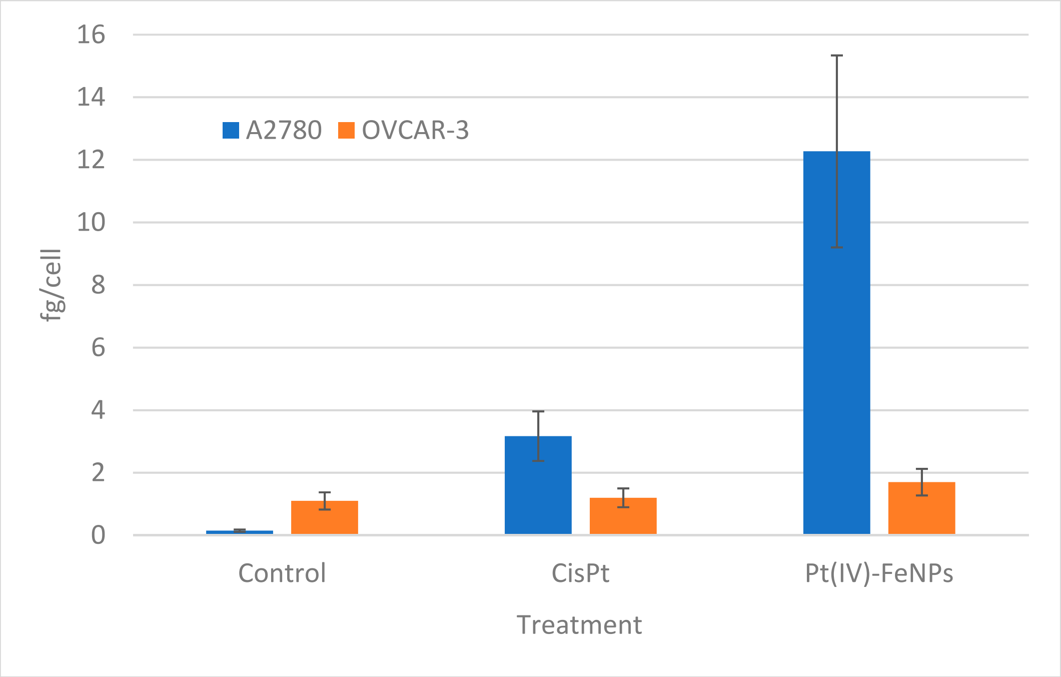

Figure S2. Incorporation results of Pt in the A2780 and OVCAR-3 exposed to cisplatin and to the Pt(IV) loaded nanoparticles.

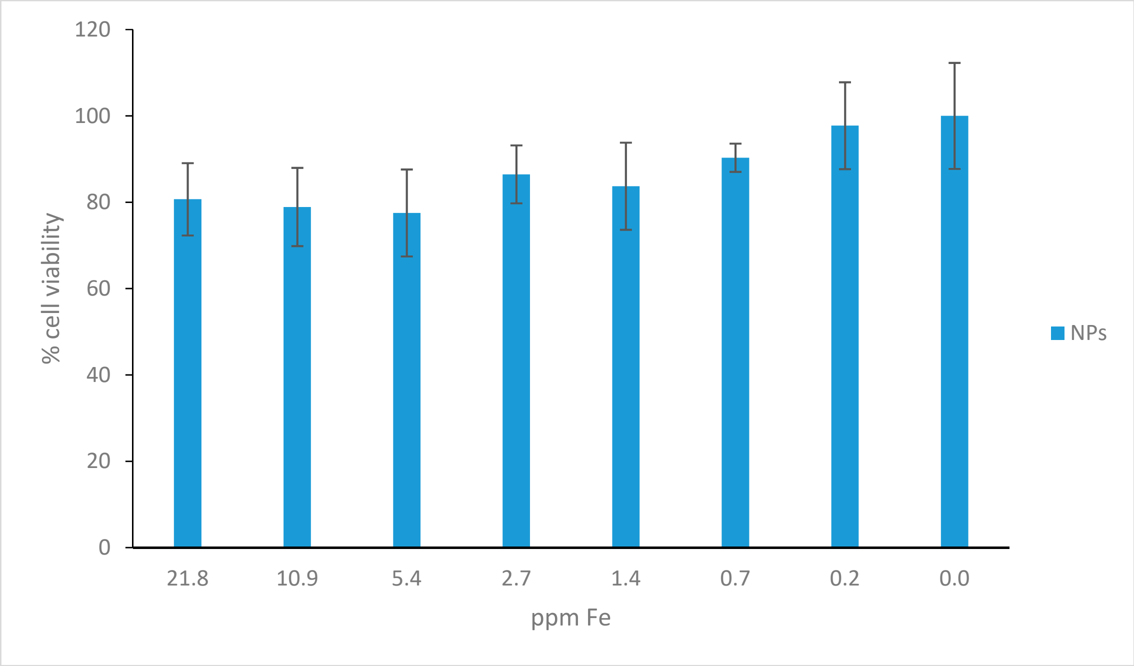

Figure S3. Viability results obtained fot the naked particles at different cocentrations after 24h exposure.

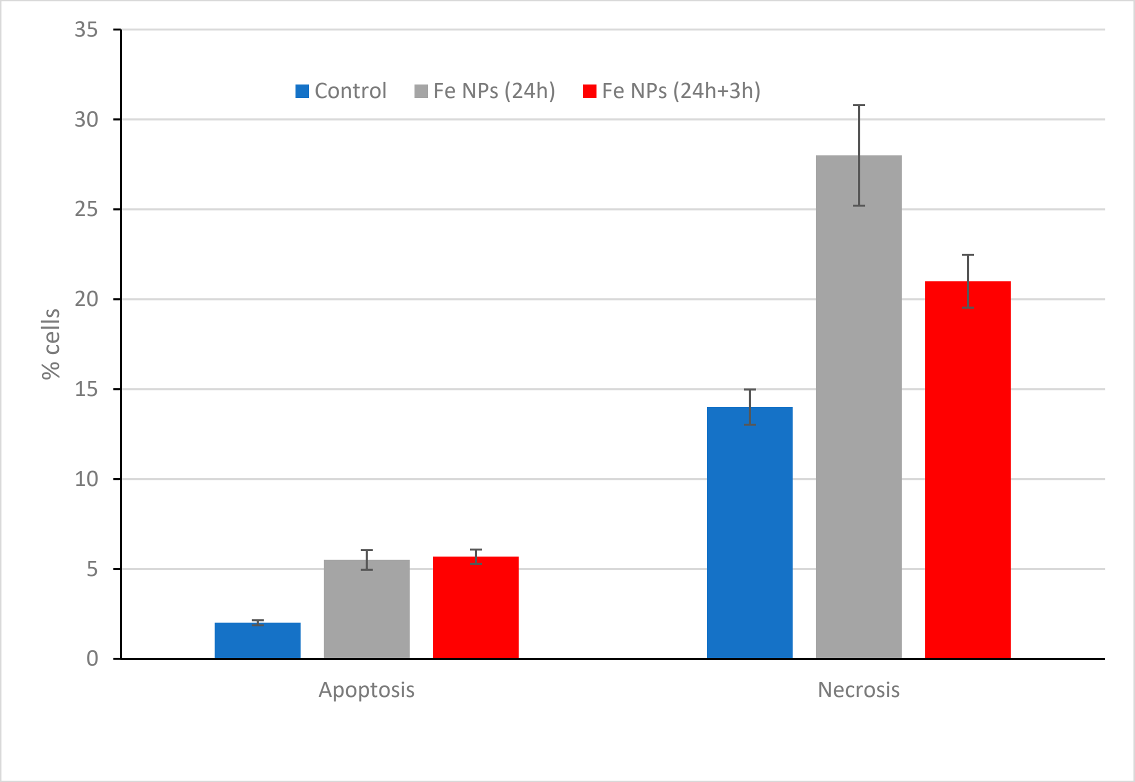

Figure S4. Apoptosis evaluated for the naked particles after 24h and 24+3 h of cell rest.

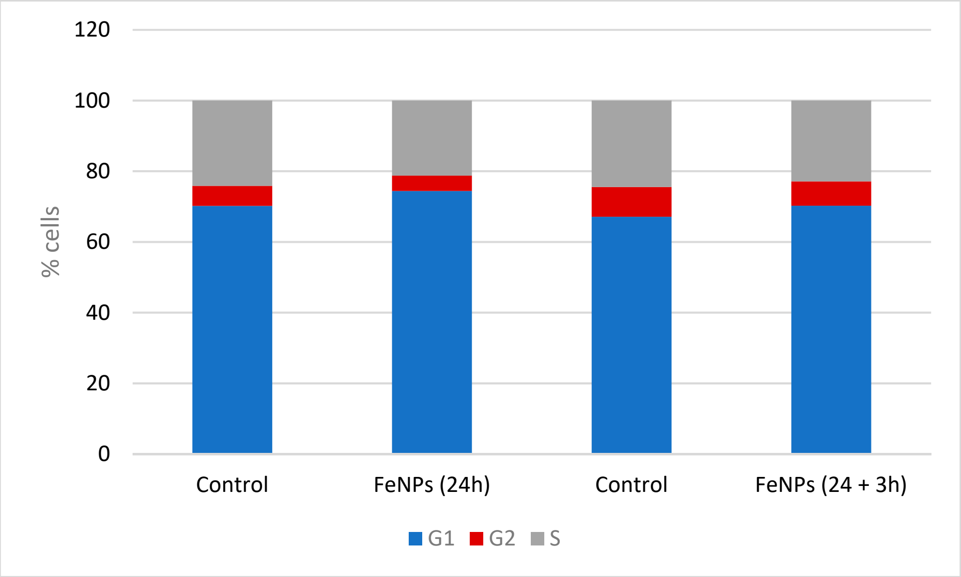

Figure S5. Cell cycle expriments for the naked particles after 24h and 24+3 h of cell rest.
